# Metronomics: Intrinsic Anakoinosis Modulator?

**DOI:** 10.3389/fphar.2018.00689

**Published:** 2018-06-25

**Authors:** André Nicolas, Manon Carré, Eddy Pasquier

**Affiliations:** ^1^Service d'Hématologie et Oncologie Pédiatrique, Hôpital pour Enfants de La Timone, AP-HM, Marseille, France; ^2^Aix-Marseille Univ., Centre National de la Recherche Scientifique, INSERM, Institut Paoli Calmettes, CRCM, Marseille, France

**Keywords:** anakoinosis, metronomic chemotherapy, drug repositioning, biology of system, chaos

Anakoinosis has been recently introduced as a global reprogramming of the communication of tumor systems, which enables conceptualizing new treatment strategies to rescue refractory tumors (Hart et al., 2015). This paradigm-shifting concept aims at revealing anticancer treatments that could perturb the communications between cellular components within tumors and in turn counteract sustained tumor growth. It is opposed to conventional chemotherapy or targeted therapies that mainly kill cancer cells, or micro-environment therapies such as anti-angiogenics (Jain et al., 2006) (i.e., anti-VEGF) or immune-modulators (Sharma and Allison, 2015) (i.e., immune checkpoint inhibitors) that focus on one given compartment of the tumor microenvironment. Instead, this new approach advocates for combinations of agents that may not exert any activity when given as single agents but which, when combined, can lead to treatment response by impacting the tumor on multiple levels in a concerted systems biological manner. Of note, agonistically active drugs are among the broad variety of regulatory active drugs that can be used for anakoinosis.

Using the metaphor of cancer as a war can help visualize this approach (Oronsky et al., 2014). “Operation overkill,” “hit hard, hit fast, and hit often” remind us about the maximum tolerated dose chemotherapy concept, and suggest that cancer is an enemy that we can defeat. However, when it comes to cancer, victory can only be achieved through total eradication. Unfortunately, in the majority of cases, in particular for metastatic and/or advanced diseases, this is not readily achievable. Moreover, this paradigm can lead to counterproductive effects leading to selection of aggressive cancer clones. Alternatively, one can imagine a guerilla strategy with repeated, coordinated ambushes, sabotages and raids that can make the invaders gradually retreat and disappear. This would be anakoinosis.

Some clinical examples of such strategies to treat refractory cancers have been reported over the years. The combination used by Reichle et al. (Thomas et al., 2015) to treat chemo-refractory acute myeloid leukemia with a combination of pioglitazone, low dose 5-azacytidine and all-trans retinoic acid, or the treatment reported by Pasquier et al. to treat angiosarcoma with daily propranolol, weekly low dose vinblastine and oral methotrexate (Pasquier et al., 2016) are illustrative examples of how this approach can be used with some success. Interestingly, these protocols can also be regarded as metronomics, which has been recently defined the combination of drug repurposing and metronomic chemotherapy (André et al., 2013).

Metronomic chemotherapy was initially defined as the frequent administration of chemotherapeutic drugs at doses significantly below the Maximum Tolerated Dose with no prolonged drug-free breaks (Kerbel and Kamen, 2004) and more recently, as the minimum biologically effective dose of a chemotherapeutic agent given as a continuous dosing regimen with no prolonged drug-free breaks that leads to anti-tumor activity (Klement and Kamen, 2011). Initially described as an antiangiogenic therapy, metronomic chemotherapy is now recognized as an intrinsic multi-targeted therapy (Pasquier et al., 2010) that can not only impact on several cellular components of the immune system (André et al., 2014) but also directly impact and cancer cells or cancer stem cells (André et al., 2017). Additionally, metronomic chemotherapy is frequently associated with drug repositioning, which consists in using already-approved medications that were not originally developed as anticancer drugs but whose antitumor properties have been unveiled (Bertolini et al., 2015).

While the definitions of metronomic is more PK grounded and anakoinosis PD grounded, they overlap greatly as anakoinosis relies on reprogramming based drug repositioning and metronomic administration of chemotherapy (vs. MTD chemotherapy) and metronomics is now regarded as a multi-targeted anticancer therapy. Whether metronomic act through forced/reprogramming behavior of cancer cells is an open debate and the term “reprogramming” needs to be better defined.

Anyhow, looking at metronomics as an anakoinosis modulator can give us interesting new insights about how this therapeutic strategy works, how can resistance occur, how future protocols should be designed, and how preclinical work could explore the full biologic impact of this approach. For instance, the acknowledgement of the multi-targeted nature of metronomics may at least in part explain the relative failure to identify reliable biomarkers when using metronomic approaches in the clinic that have mainly focused on angiogenic biomarkers. Elsewhere, the complex combinations of anti-cancer agents with metronomic etoposide, cyclophosphamide, thalidomide, fenofibrates, celecoxib, bevacizumab and intrathecal aracytine and etoposide have been showed to induce long-term remission or tumor control in refractory medulloblastoma or atypical rhabdoid terratoid tumors in children (Peyrl et al., 2012), while none of the agent used can lead to such effects when used as monotherapy. Interestingly, a similar combination without thalidomide also led to tumor control in a primary refractory atypical rhabdoid terratoid tumor (Berland et al., 2017), illustrating that at the individual level, not all drugs are necessary to ensure clinical activity. However, the global combination of agents would likely ensure activity in a majority of patients through simultaneous targeting of important interacting molecular pathways and hubs resulting in a “biologic system” targeting effect.

In fact, the concept of anakoinosis is similar in some ways to the chaotic perspective of cancer (Coffey, 1998). Researchers and clinicians have focused on breaking down tumors into individual (cells) and sub-individual elements (chromosomes, genes, mutations) with ever-increasing details. However, one of the major resulting consequence is that extrapolation from individual components behavior to the behavior of the whole system is not reliable, mostly because non-linear interactions between the components render the system chaotic (Weiss et al., 1994; Janecka, 2007). Moreover, at the outer edge of chaos, communications between cancer cells and the system is altered, and cancer cells grow at the expense of the larger system (Janecka, 2007)

Actually, the best strategies to control a chaotic system rely on the introduction of small perturbations administered with good timing (Coffey, 1998). Therefore, by relying on the frequent administration of agents at relatively low dose, metronomics introduces small perturbations in the chaotic system and may therefore represent a promising new approach to cancer treatment. Similarly, as proposed by Janecka (Janecka, 2007), treating a chaotic complex cancer system shall not rely on cellular killing but on cellular “retraining”.

The recent introduction of the concept of anakoinosis unveils a new rationale to treat cancer as well as a new frame to potentially understand the complex pharmaco-dynamics of metronomics related to its intertwined biological effects.

Most studies have focused on metronomic chemotherapy as an anti-angiogenic strategy, as a pro-immune strategy or more rarely as an anticancer cells strategy (André et al., 2017; Orlandi et al., 2018). Metronomic chemotherapy has almost never been considered to impact/reprogram a whole tumor system at several levels. Considering, metronomic as an anakoinosis modulator introduces a paradigm shift which could pave the way for better understanding and using it.

## Author contributions

All authors contributed the concept of this opinion article, wrote, edited the drafts, and final version of the manuscript.

### Conflict of interest statement

The authors declare that the research was conducted in the absence of any commercial or financial relationships that could be construed as a potential conflict of interest.
